# Supplementary material for: Coordinated regulation of vegetative phase change by brassinosteroids and the age pathway in Arabidopsis
Source: Nat Commun. 2023 May 5;14:2608. doi: 10.1038/s41467-023-38207-z (PMC10163027; doi:10.1038/s41467-023-38207-z)
Supplement: Supplementary file 1 — Supplementary Information [file 41467_2023_38207_MOESM1_ESM.pdf]

Supplementary Figures and Supplementary Tables

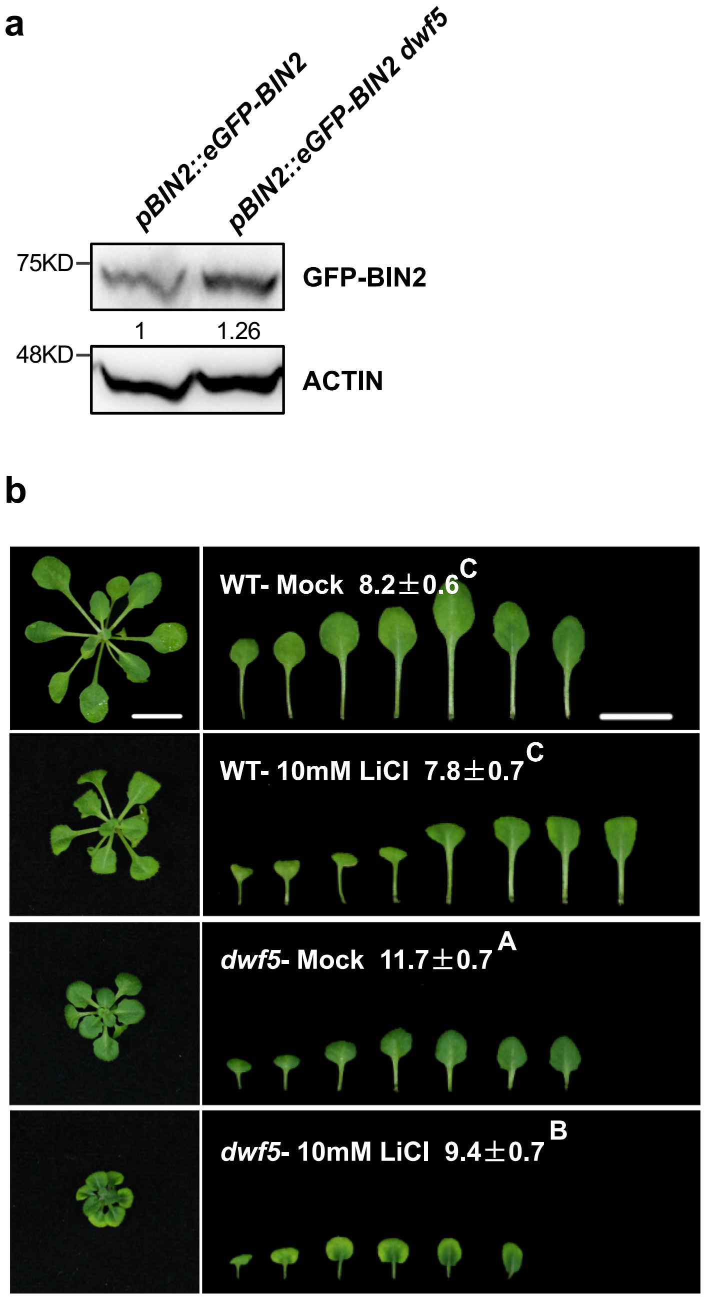

Supplementary Fig. 1 Elevated levels of BIN2 in *dwf5* contribute to the delayed vegetative phase change phenotype of *dwf5*. a The BIN2 protein level is elevated in *dwf5*. Total protein was extracted from 15-day-old plants *pBIN2::eGFP-BIN2* and *pBIN2::eGFP-BIN2 dwf5*, and separated in a SDS-PAGE gel. The blot was then detected by Western blotting using an anti-GFP

antibody and an anti-ACTIN antibody, respectively. Numbers between two blots indicate the relative normalized value for each sample. The intensity of each sample was first normalized to its corresponding ACTIN, then the resultant value was normalized again to the value of *pBIN2::eGFP-BIN2*. The band intensity was determined using Image J. All experiments were repeated 3 times biologically with similar results. **b** Phenotype of 30-day-old WT and *dwf5* plants. WT and *dwf5* plants were grown on 1/2 MS medium containing 0 or 10 mM LiCl in short days. Numbers indicate the first leaf with abaxial trichome (n=20, ±SD). Different letters indicate significant difference between genotypes using one-way ANOVA at P<0.01. Scale bar = 1 cm.

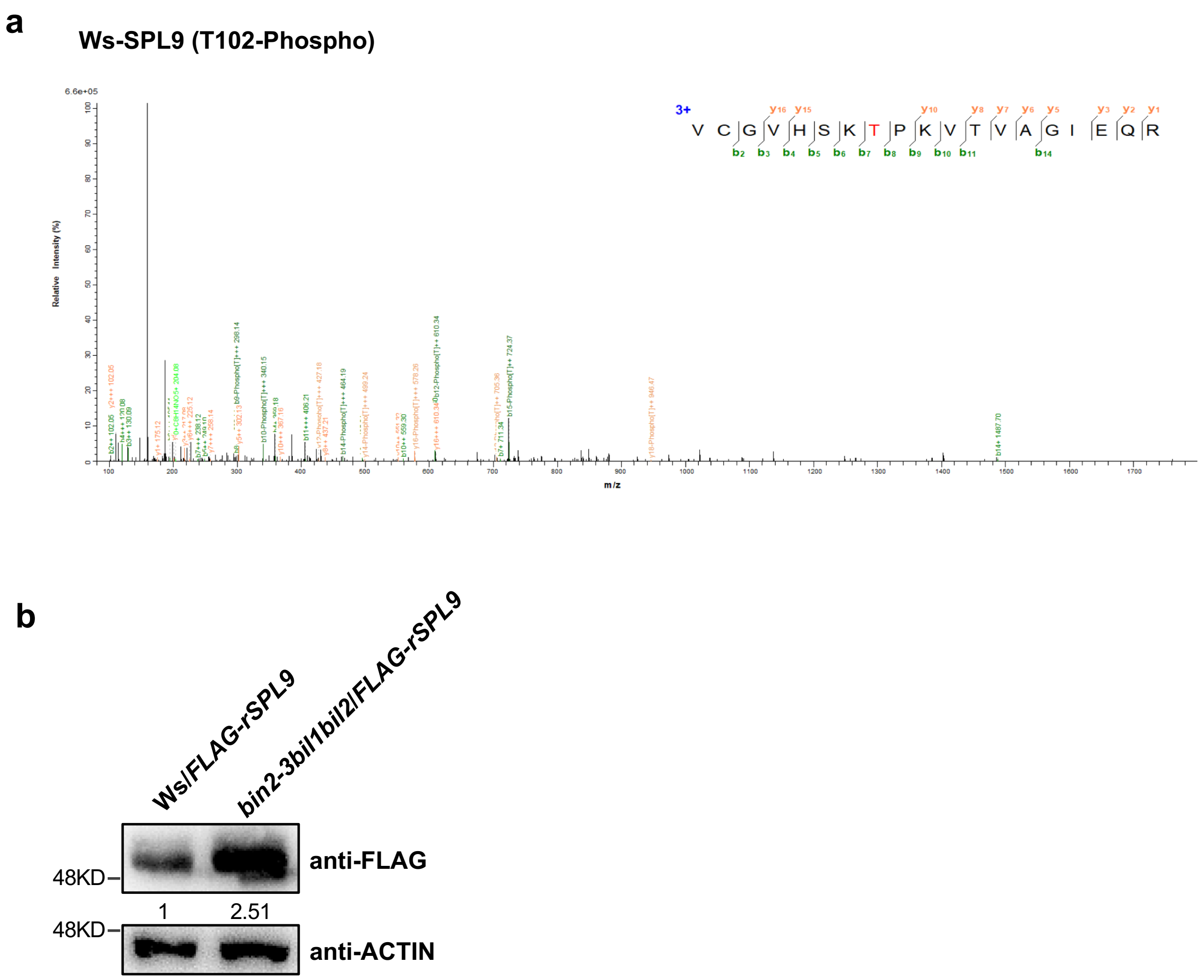

Supplementary Fig. 2 LC-MS/MS analysis of SPL9 and the level of SPL9 in *bin2-3bil1bil2*. a LC-MS/MS analysis of 3×FLAG-rSPL9 in Ws and *bin2-3bil1bil2*. A phosphopeptide of FLAG-rSPL9 indicates BIN2 phosphorylation sites in SPL9 at T102 (VCGVHSK**T**PKVTVAGIEQR). **b** SPL9 is over-accumulated in *bin2-3 bil1 bil2*. Numbers between two blots indicate the relative normalized value for each sample. The intensity of each sample was first normalized to its corresponding ACTIN, then the resultant value was normalized again to the value of Ws. The band intensity was determined using Image J. LC-MS/MS analysis was performed once, Western blotting were repeated 3 times biologically with similar results.

a

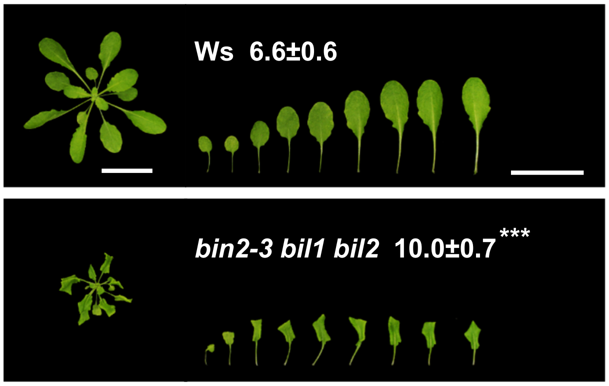

b

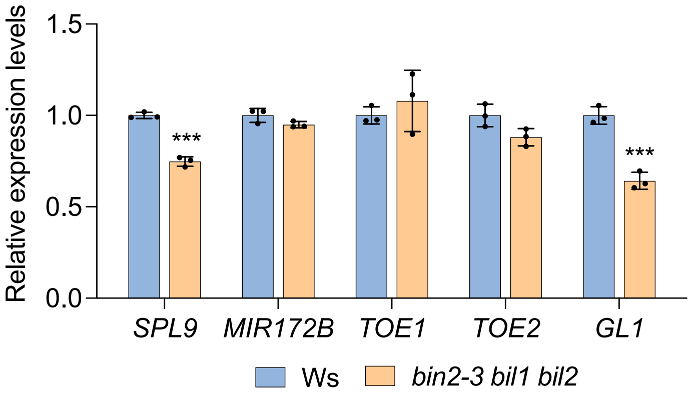

Supplementary Fig. 3 Phenotypic characterization and gene expressions in Ws and *bin2-3bil1bil2*. a Phenotypic characterization of WT and *bin2-3bil1bil2*. 34-day-old plants were harvested for phenotypic analysis. Numbers indicate the first leaf with abaxial trichomes (n=25 plants, ±SD). Asterisks denote significant difference from Ws using two-tailed Student's t-test (P<0.001). Scale bar = 1 cm. All experiments were repeated 3 times biologically. **b** The expression levels of *SPL9*, *MIR172B*, *TOE1*, *TOE2* and *GL1* in 12-day-old Ws and *bin2-3 bil1 bil2* in short days. Data are means ±SD from a representative experiment with three technical replicates for each sample. Asterisk denotes significant difference from WT using two-tailed Student's t-test at P<0.001.

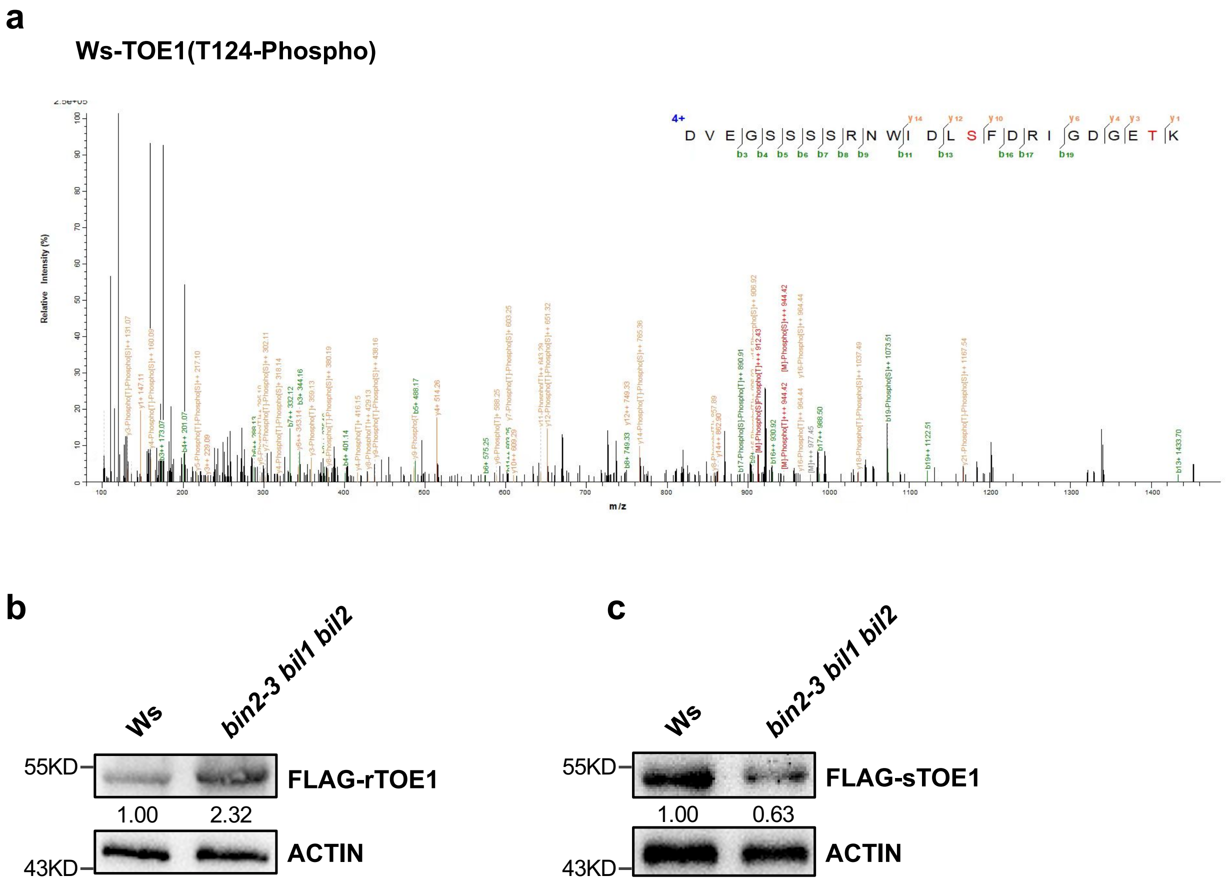

Supplementary Fig. 4 LC-MS/MS analysis of TOE1 and the level of TOE1 in *bin2-3bil1bil2*. **a** LC-MS/MS analysis of 3×FLAG-rTOE1 in Ws and *bin2-3bil1bil2*. A phosphopeptide of FLAG-rTOE1 indicates BIN2 phosphorylation sites in TOE1 at T124 (DVEGSSSSrNWIDLSFDRIGDGE**T**K). **b, c** 3×FLAG-rTOE1 is elevated in *bin2-3bil1bil2* (b), but 3×FLAG-sTOE1 is reduced (c). Numbers between two blots indicate the relative normalized value for each sample. The intensity of each sample was first normalized to its corresponding ACTIN, then the resultant value was normalized again to the value of Ws. The band intensity was determined using Image J. LC-MS/MS analysis was performed once, Western blotting were repeated 3 times biologically with similar results.

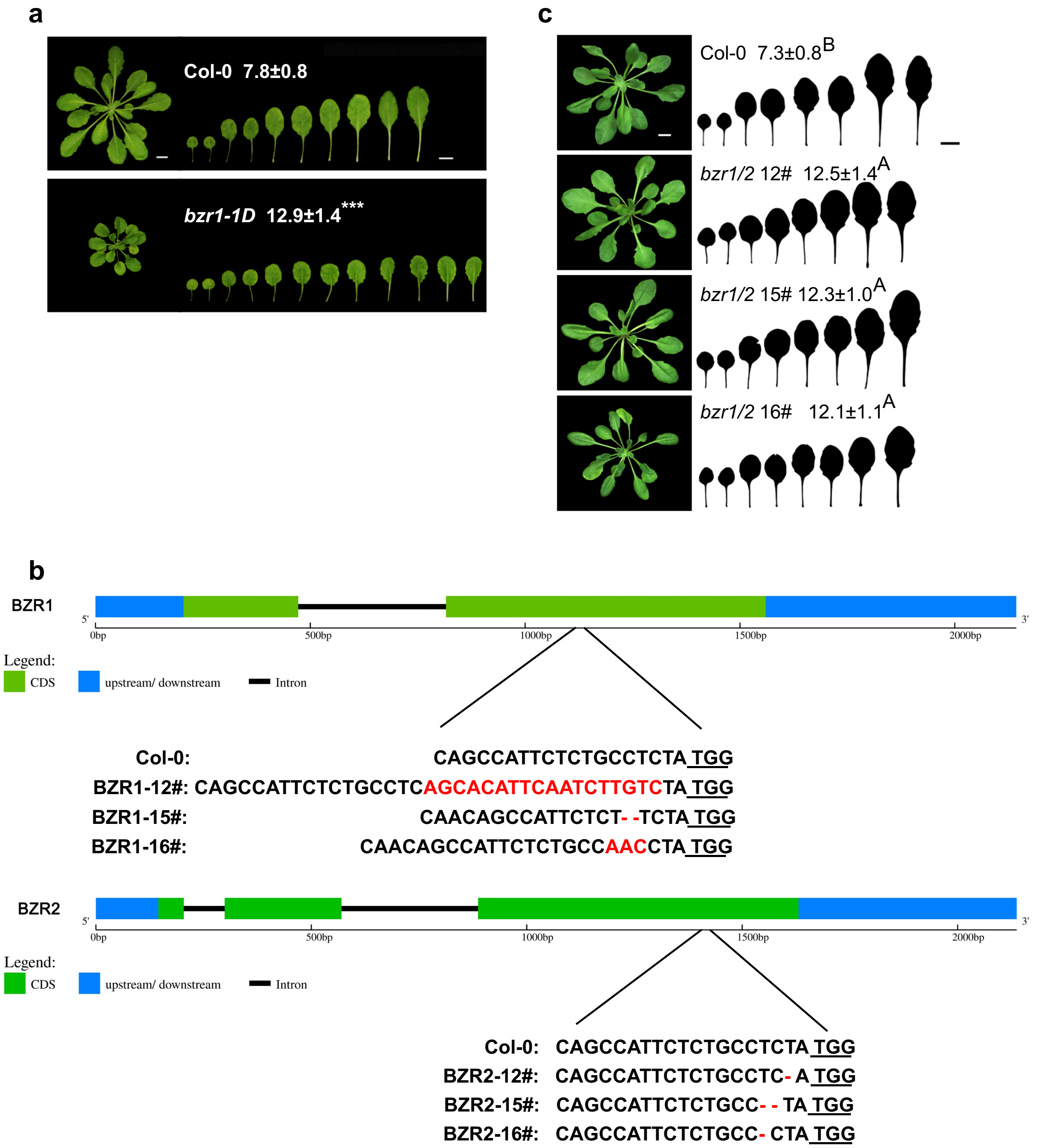

Supplementary Fig. 5 Phenotypic characterization of *bzr1-1D* and *bzr1/2*. **a** Phenotype of 34-day-old Col-0 and *bzr1-1D* plants in short days. Numbers indicate the first leaf with abaxial trichomes (*n*=25 plants, ±SD). Asterisk denotes significant difference from WT using two-tailed Student's *t*-test at *P*<0.001. Scale bar = 1 cm. **b** Schematic diagram of the *BZR1/2* gene structure and the Cas9-edited DNA sequence in *BZR1/2*. A common gRNA targeting both *BZR1/2* was

designed to edit the sequence. **c** Phenotype of 20-day-old Col-0 and *bzr1/2* plants grown in short days. Numbers indicate the first leaf with abaxial trichomes (*n*=15 plants, ±SD). Different letters indicate significant difference between genotypes using one-way ANOVA at *P*<0.001. Scale bar = 1 cm.

Supplementary Table Primers used in this study

**Primers for Genotyping**		
**Name**	**Primers**	**Sequence (5' to 3')**
spl9-4	SAIL_150_B05-LP	TGGTTCCTCCACTGAGTCATC
	SAIL_150_B05-LP	GCTCATTATGACCAGCGAGTC
	LB1	GCCTTTTCAGAAATGGATAAATAGCCTTGCTTCC
toe1	SALK_069677-LP	TAGGTGGTTTCGACACTGCTC
	SALK_069677-RP	CGAGGATCCATAAGGAAGAGG
toe2	SALk_065370-LP	GAATTTGACAGCAGCTCGATC
	SALk_065370-RP	TGTTTATTTCAAATCGACGGC
	LBb1.3	ATTTTGCCGATTTCGGAAC
dwf5	dwf5-dCaps-EcoRI-F	AGAGCTTCACCTCTTTCTGATT
	dwf5-dCaps-EcoRI-R	ATGTGCAATGTCCATGGAATT
bzr1-1D	bzr1- dCAPA-	GCCTAACTGGGAATCTATCG

	SmaI-F	
	bzr1- dCAPS-SmaI-R	CACATTCAGGTATAGTACCC
For sequencing	F11F12(ZBY)-F	ATGGCGGAGACTGTACATTCTCCG
	F11F12(ZBY)-R	TCAATAAATTCCCGGAATGATCCTG
**Primers for constructs**		
**Constructs**	**Primers**	**Sequence (5' to 3')**
pDWF5::DWF5	DWF5-KpnI-genomic-F	CGGGGTACCTCCTTCCTCCACAACACGAT
	DWF5-KpnI-genomic-R	CGGGGTACCGTCCAAACACGCCCTAACAA
pGADT7-BIN2	BIN2-EcoRI-F	CCGGAATTCATGGCTGATGATAAGGAG
	BIN2-BamHI-R	CGGGATCCTTAAGTTCCAGATTGATTC
pGBKT7-SPL9	SPL9-Nde1-F	GGGAATTCCATATGATGGAGATGGGTTCCAACTC
	SPL9-EcoR1-R	CCGGAATTCTCAGAGAGACCAGTTGGTAT
pGADT7-TOE1	TOE1-AD-F	GTACCAGATTACGCTCATATGATGTTGGATCTTAACCTCAACGC
	TOE1-AD-R	CAGCTCGAGCTCGATGGATCCTTAAGGGTGTGGATAAAAGTAACCACG
pXY103-SPL9 (nYFP)	SPL9-103/105-XbaI-F	GCTCTAGAATGGAGATGGGTTCCAA
	SPL9-103-SalI-R	ACGCGTCGACGAGAGACCAGTTGGTA
pXY103-TOE1	TOE1-nYFP-F	ATTACAGGTACCCGGGGATCCATGTTGGATCTTAACCTCA

(nYFP)		ACGC
	TOE1-nYFP-R	GCTCACCATACCGCCGTCGACAGGGTGTGGATAAAAGTAACCACG
pXY105-BIN2 (cYFP)	BIN2-BamH1-F	CGGGATCCATGGCTGATGATAAGGAG
	BIN2-Sal1-105-R	ACGCGTCGACTTAAGTTCCAGATTGATTC
pET30a(+)-SPL9 (6×His)	SPL9-EcoRV-F	TTGGATATCATGGAGATGGGTTCCAACTCGG
	SPL9-EcoR1-R(-TGA)	CCGGAATTCGAGAGACCAGTTGGTATGGTG
pET30a(+)-TOE1 (6×His)	TOE1-30a-F	GCCATGGCTGATATCGGATCCATGTTGGATCTTAACCTCAACGC
	TOE1-30a-R	TGCGGCCGCAAGCTTGTCGACAGGGTGTGGATAAAAGTAACCACG
pPGH-BIN2 (GST)	BIN2-inphu-BamH1-F	GGTTCCGCGTGGATCCATGGCTGATGATAAGGAG
	BIN2-inphu-Sal1-R	GGCCGCTCGAGTCGACTTAAGTTCCAGATTGATTC
35S::3×FLAG-rSPL9	3×Flag-NcoI-F	CATGCCATGGACTACAAGGACCACGACGG
	SPL9-R1	ACGCAGGCTGTGGCTTCCTTCGTCGCTCA
	SPL9-F2	TGAGCGACGAAGGAAGCCACAGCCTGCGT
	SPL9-R	TCAGAGAGACCAGTTGGTATGGTGAG
35S::BIN2-GFP	BIN2-BamH1-F	CGGGATCCATGGCTGATGATAAGGAG
	BIN2-Sal1-R(-TGA)	ACGCGTCGACAGTTCCAGATTGATTC
35S::BIN2-MYC	BIN2-CoIP-F1	AACACGGGGGACTTTGCAACATGGCTGATGATAAGGAG

		ATGCCTGC
	BIN2-CoIP-R1	TCAAACCCATAGTTCCAGATTGATTCAAGAAGCTTAGACCC
	BIN2-CoIP-F2	ATCTGGAACTATGGGTTTGATTAACGGTGAACAGAAACTGATTTC
	BIN2-CoIP-R2	TGAAGACAGAGCTAGTTACATTAGGAGCCATTCAAATCCTCTTCCGAG
35S::3×HA-rTOE1	TOE1-CoIP-F1	AACACGGGGGACTTTGCAACATGTTGGATCTTAACCTCAACGCTGATTCTC
	TOE1-CoIP-R1	CGTATGGGTAAGGGTGTGGA
	TOE1-CoIP-F2	TCCACACCCTTACCCATACG
	TOE1-CoIP-R2	TGAAGACAGAGCTAGTTACATTAAGCGTAATCTGGAACGTCATATGGATAGG
pSPL9::3×FLAG-rSPL9-AXXXA	SPL9-mut- F1	GGTGGTGGATCCGGGTCTTCTTCCT
	SPL9-mut-TA-R1	AGCGACTTTAGGTGCTTTAGAGTGCACTCCACAAAC
	SPL9-mut-TA-F2	CTAAAGCACCTAAAGTCGCTGTGGCTGGTATCGAACAG
	SPL9-NcoI-R	CATGCCATGGTTACAAGAATCATACGGAAATTCATG
pSPL9::3×FLAG-rSPL9-DXXXD	SPL9-TD-R1	ATCGACTTTAGGGTCTTTAGAGTGCACTCCACAAAC
	SPL9-TD-F2	CTAAAGACCCTAAAGTCGATGTGGCTGGTATCGAACAG
Ubi10::3×FLAG-rTOE1	PSY06-3×FLAG-F1	TATCGATGGCGCCAGCTGCAG ATGGACTACAAGGACCACGAC
	3×FLAG-TOE1-R1	GCGTTGAGGTTAAGATCCAACATCTTGTCATCGTCATCCTTGT

	TOE1-F1	ATGTTGGATCTTAACCTCAACGC
	PSY06-TOE1-R2	CCGGGCCCTATATATGGATCCTTAAGGGTGTGGATAAAAGTAACCACG
Ubi10::3×FLAG-rTOE1-AXXXT	TOE1-TA-R1	AGTTACCAATTTAGCTTCTCCGTCACC
	TOE1-TA-F2	GGTGACGGAGAAGCTAAATTGGTAACTCCGGTTCCGACTCCGGCT
Ubi10::3×FLAG-rTOE1-DXXXT	TOE1-TD-R1	AGTTACCAATTTATCTTCTCCGTCACC
	TOE1-TD-F2	GGTGACGGAGAAGATAAATTGGTAACTCCGGTTCCGACTCCGGCT
pBIN2::eGFP-BIN2	pBIN2-Kpn1-F	CGGGGTACCGGTTCAGTGGTCTGGATTCTCG
	pBIN2-Nco1-R	CTCACCATGGCGATAGAGACACAGAGAGG
	eGFP-Nco1-F	ATCGCCATGGTGAGCAAGGGCGAGGAG
	Overlap-eGFP-link-R	CATGGACCTGATAGCGGCGTTCTTGTACAGCTCGTCCATGC
	Overlap-link-BIN2-CDS-F	AACGCCGCTATCAGGTCCATGATGGCTGATGATAAGGAGATGC
	BIN2-CDS-Pml1-R	TTAAGTTCCAGATTGATTCAAGAAGCTTAGACCC
Cas9-BZR1/2	Cas9-BZR1/2-F	ATTGCAGCCATTCTCTGCCTCTA
	Cas9-BZR1/2-R	AAACTAGAGGCAGAGAATGGCTG
**Primers for qRT-PCR**		
	**Primers**	**Sequences (5' to 3')**
**Primers for**	miR156-RT	GTCGTATCCAGTGCAGGGTCCGAGGTATTCGCACTGGAT

**miRNA real-time PCR**		ACGACGTGCTC
	miR172-RT	GTCGTATCCAGTGCAGGGTCCGAGGTATTCGCACTGGATACGACATGCAG
	miR156-F	GCGGCGGTGACAGAAGAGAGT
	miR172-F	GCGGCGGAGAATCTTGATGATG
	Universal-R	GTGCAGGGTCCGAGGT
	snoR101-F	CTTCACAGGTAAGTTCGCTTG
	snoR101-R	AGCATCAGCAGACCAGTAGTT
**Primers for real-time PCR**	qTUB2-F	GAGCCTTACAACGCTACTCTGTCTGTC
	qTUB2-R	ACACCAGACATAGTAGCAGAAATCAAG
	qMIR172B-F	TTTCTCAAGCTTTAGGTATTTGTAG
	qMIR172B-R	TCGGCGGATCCATGGAAGAAAGCTC
	qSPL3-F	ATGAGTATGAGAAGAAGCAAAGCG
	qSPL3-R	TCCACTACTACTTGTAGCTTTACCT
	qSPL9-F	GGAATTTGACCTAGAGAAAAGGAGTT
	qSPL9-R	GCATCACCATTTTCGTAAAGCGAAG
	qSPL13-F	GGGTTTTCAAGGTAGCAAATTGCT
	qSPL13-R	ACCAACAACATAGCTCTGGCTCTG
	qTOE1-F	CGAGTTATAATAATCCCGCCGAG
	qTOE1-R	TTAAGGGTGTGGATAAAAGT
	qTOE2-F	ATGGAGAACCACATGGCTGC
	qTOE2-R	GGTGCTGTAGCTGCTACGGC
	qGL1-F	TGGAACCGCATCGTCAGA
	qGL1-R	ATTGCCGAGGAGCTTGTG
