# Supplementary material for: Coordinated regulation of vegetative phase change by brassinosteroids and the age pathway in Arabidopsis
Source: Nat Commun. 2023 May 5;14:2608. doi: 10.1038/s41467-023-38207-z (PMC10163027; doi:10.1038/s41467-023-38207-z)
Supplement: Supplementary file 3 — Reporting Summary [file 41467_2023_38207_MOESM3_ESM.pdf]

Corresponding author(s): Shengjun Feng & Gang Wu

Last updated by author(s): Apr 11, 2023

Reporting Summary

Nature Portfolio wishes to improve the reproducibility of the work that we publish. This form provides structure for consistency and transparency in reporting. For further information on Nature Portfolio policies, see our Editorial Policies and the Editorial Policy Checklist.

Statistics

For all statistical analyses, confirm that the following items are present in the figure legend, table legend, main text, or Methods section.

n/a	Confirmed	
☐	☒	The exact sample size (*n*) for each experimental group/condition, given as a discrete number and unit of measurement
☐	☒	A statement on whether measurements were taken from distinct samples or whether the same sample was measured repeatedly
☐	☒	The statistical test(s) used AND whether they are one- or two-sided<br>*Only common tests should be described solely by name; describe more complex techniques in the Methods section.*
☒	☐	A description of all covariates tested
☒	☐	A description of any assumptions or corrections, such as tests of normality and adjustment for multiple comparisons
☐	☒	A full description of the statistical parameters including central tendency (e.g. means) or other basic estimates (e.g. regression coefficient) AND variation (e.g. standard deviation) or associated estimates of uncertainty (e.g. confidence intervals)
☒	☐	For null hypothesis testing, the test statistic (e.g. *F*, *t*, *r*) with confidence intervals, effect sizes, degrees of freedom and *P* value noted<br>*Give P values as exact values whenever suitable.*
☒	☐	For Bayesian analysis, information on the choice of priors and Markov chain Monte Carlo settings
☒	☐	For hierarchical and complex designs, identification of the appropriate level for tests and full reporting of outcomes
☒	☐	Estimates of effect sizes (e.g. Cohen's *d*, Pearson's *r*), indicating how they were calculated

Our web collection on statistics for biologists contains articles on many of the points above.

Software and code

Policy information about availability of computer code

Data collection	qRT-PCR experiments were performed using StepOnePlus Real-Time System. Western blot analysis were permormed using the Tanon 5200Multi gel documentation system.
Data analysis	All graphs were generated using Microsoft Excel 2016. qRT-PCR data analysis was carried out according to Livak and Schmittgen, (2001) (For miRNA analyses) and Feng et al. (2016) (For mRNA analyses).

For manuscripts utilizing custom algorithms or software that are central to the research but not yet described in published literature, software must be made available to editors and reviewers. We strongly encourage code deposition in a community repository (e.g. GitHub). See the Nature Portfolio guidelines for submitting code & software for further information.

Data

Policy information about availability of data

All manuscripts must include a data availability statement. This statement should provide the following information, where applicable:

- Accession codes, unique identifiers, or web links for publicly available datasets
- A description of any restrictions on data availability
- For clinical datasets or third party data, please ensure that the statement adheres to our policy

Provide your data availability statement here.

Research involving human participants, their data, or biological material

Policy information about studies with human participants or human data. See also policy information about sex, gender (identity/presentation), and sexual orientation and race, ethnicity and racism.

Reporting on sex and gender	*Use the terms sex (biological attribute) and gender (shaped by social and cultural circumstances) carefully in order to avoid confusing both terms. Indicate if findings apply to only one sex or gender; describe whether sex and gender were considered in study design; whether sex and/or gender was determined based on self-reporting or assigned and methods used.* *Provide in the source data disaggregated sex and gender data, where this information has been collected, and if consent has been obtained for sharing of individual-level data; provide overall numbers in this Reporting Summary. Please state if this information has not been collected.* *Report sex- and gender-based analyses where performed, justify reasons for lack of sex- and gender-based analysis.*
Reporting on race, ethnicity, or other socially relevant groupings	*Please specify the socially constructed or socially relevant categorization variable(s) used in your manuscript and explain why they were used. Please note that such variables should not be used as proxies for other socially constructed/relevant variables (for example, race/ethnicity should not be used as a proxy for socioeconomic status).* *Provide clear definitions of the relevant terms used, how they were provided (by the participants/respondents, the researchers, or third parties), and the method(s) used to classify people into the different categories (e.g. self-report, census or administrative data, social media data, etc.)* *Please provide details about how you controlled for confounding variables in your analyses.*
Population characteristics	*Describe the covariate-relevant population characteristics of the human research participants (e.g. age, genotypic information, past and current diagnosis and treatment categories). If you filled out the behavioural & social sciences study design questions and have nothing to add here, write "See above."*
Recruitment	*Describe how participants were recruited. Outline any potential self-selection bias or other biases that may be present and how these are likely to impact results.*
Ethics oversight	*Identify the organization(s) that approved the study protocol.*

Note that full information on the approval of the study protocol must also be provided in the manuscript.

Field-specific reporting

Please select the one below that is the best fit for your research. If you are not sure, read the appropriate sections before making your selection.

[x] Life sciences [ ] Behavioural & social sciences [ ] Ecological, evolutionary & environmental sciences

For a reference copy of the document with all sections, see nature.com/documents/nr-reporting-summary-flat.pdf

Life sciences study design

All studies must disclose on these points even when the disclosure is negative.

Sample size	The authors did not use statistical method to predetermine sample size. The sample sizes are standard for this type of experiments and the reproducibility between independent experiments warrants sample sizes to be sufficient.
Data exclusions	No data were intentionally excluded.
Replication	Al experiments were repeated three times with similar results except that the analysis of the concentration of brassinolide (BL) and the mass spectrometry analysis of phosphorylation site were only performed once.
Randomization	Plants used for experiments were grown randomly in the same growth chamber to avoid positional effects, and allocated to groups based on genotype and treatment.
Blinding	The authors were not blinded during this study, which would have been difficult due to the obvious phenotypes of the genetic material. Most of the results are quantitative, or not subjective.

Reporting for specific materials, systems and methods

We require information from authors about some types of materials, experimental systems and methods used in many studies. Here, indicate whether each material, system or method listed is relevant to your study. If you are not sure if a list item applies to your research, read the appropriate section before selecting a response.

Materials & experimental systems

n/a	Involved in the study
☐	☒ Antibodies
☒	☐ Eukaryotic cell lines
☒	☐ Palaeontology and archaeology
☒	☐ Animals and other organisms
☒	☐ Clinical data
☒	☐ Dual use research of concern
☒	☐ Plants

Methods

n/a	Involved in the study
☒	☐ ChIP-seq
☒	☐ Flow cytometry
☒	☐ MRI-based neuroimaging

Antibodies

Antibodies used

Antibodies used for CoIP : Myc-Tag Rabbit mAb (ABclonal, AE070); Rabbit anti HA-Tag pAb (ABclonal, AE036); Purified immunoglobulin, buffered aqueous glycerol solution（Millipore, A2220）.
Antibodies used for Pull down: Anti-6×His Tag mouse monoclonal antibody (Sangon Biotech, D191001); ProteinFind® Anti-GST Mouse Monoclonal Antibody (TransGen, HT601-01)
Antibodies used for WB: Mouse anti DDDDK-Tag mAb (ABclonal, AE005); anti-FLAG (Beyotime, AF519); Anti-ACTIN(Plants) rabbit polyclonal antibody (Sangon Biotech, D110007); Anti-GFP rabbit polyclonal antibody (Sangon Biotech, D110008)

Validation

Have been reported in previous study (Zhou et al, 2016/PMID:27352805; Pei et al, 2021/PMID: 34953386; Huang et al, 2022/PMID: 35085106; Cao et al, 2020/PMID: 32422188; Li et al, 2020/PMID: 31706031)
