# Supplementary material for: Coordinated regulation of vegetative phase change by brassinosteroids and the age pathway in Arabidopsis
Source: Nat Commun. 2023 May 5;14:2608. doi: 10.1038/s41467-023-38207-z (PMC10163027; doi:10.1038/s41467-023-38207-z)

**Figure 2c**


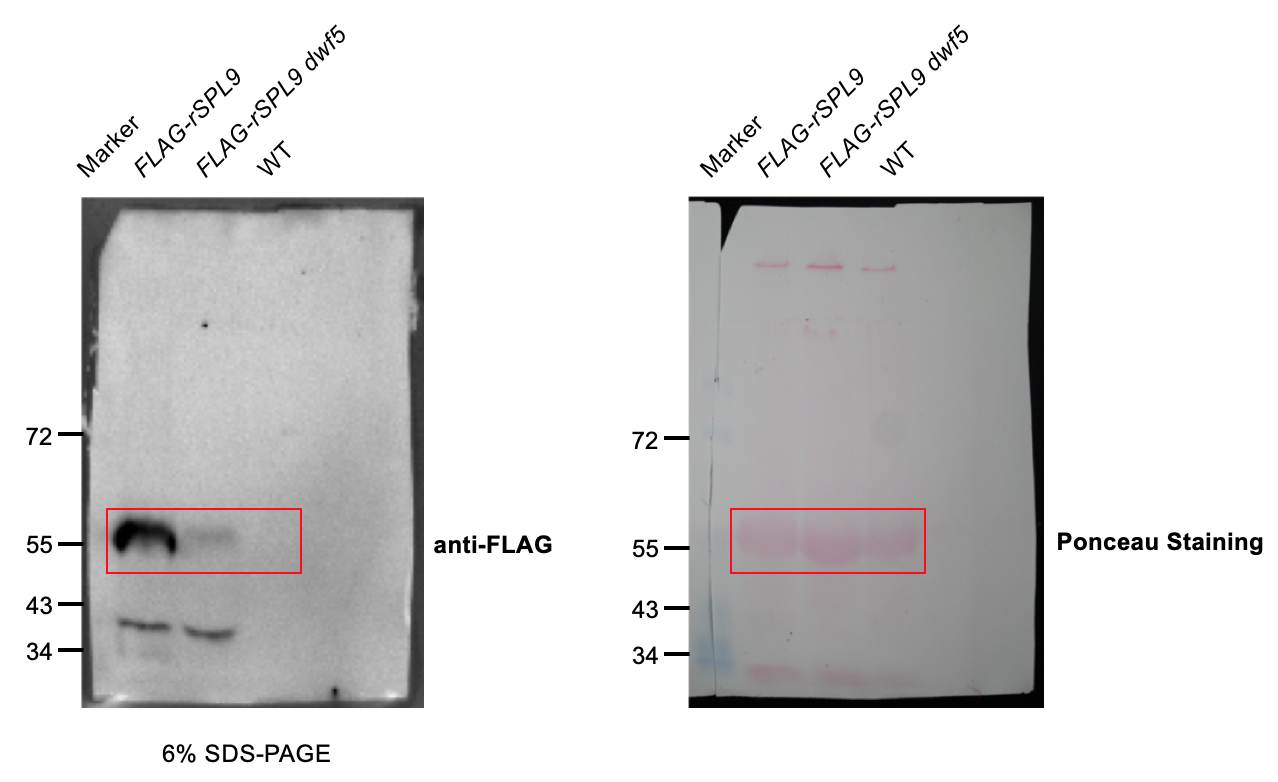


**Figure 2e**


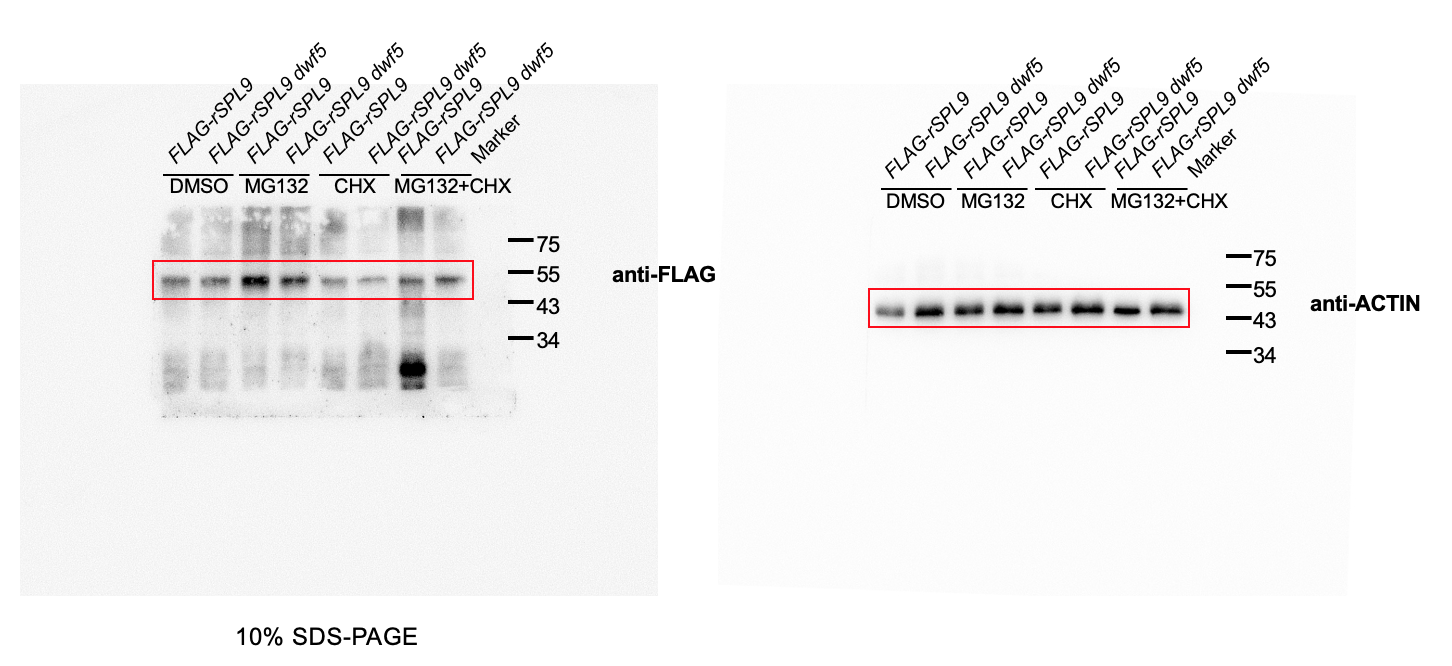


**Figure 2f**


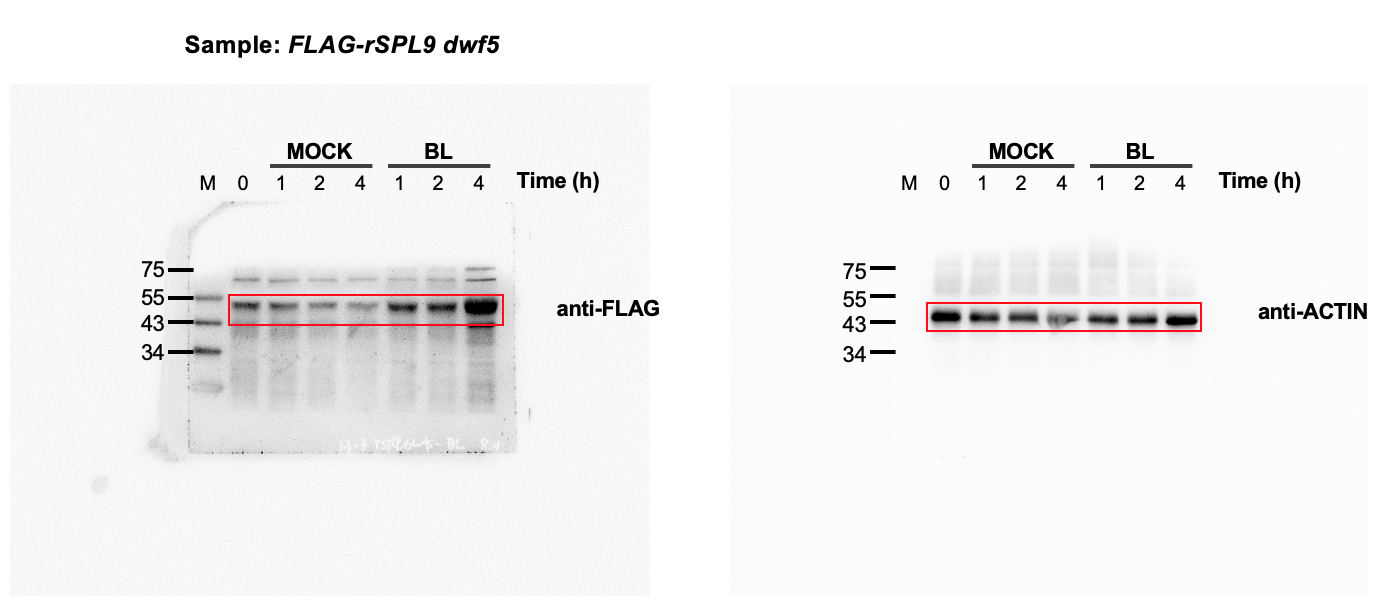


**Figure 4c**


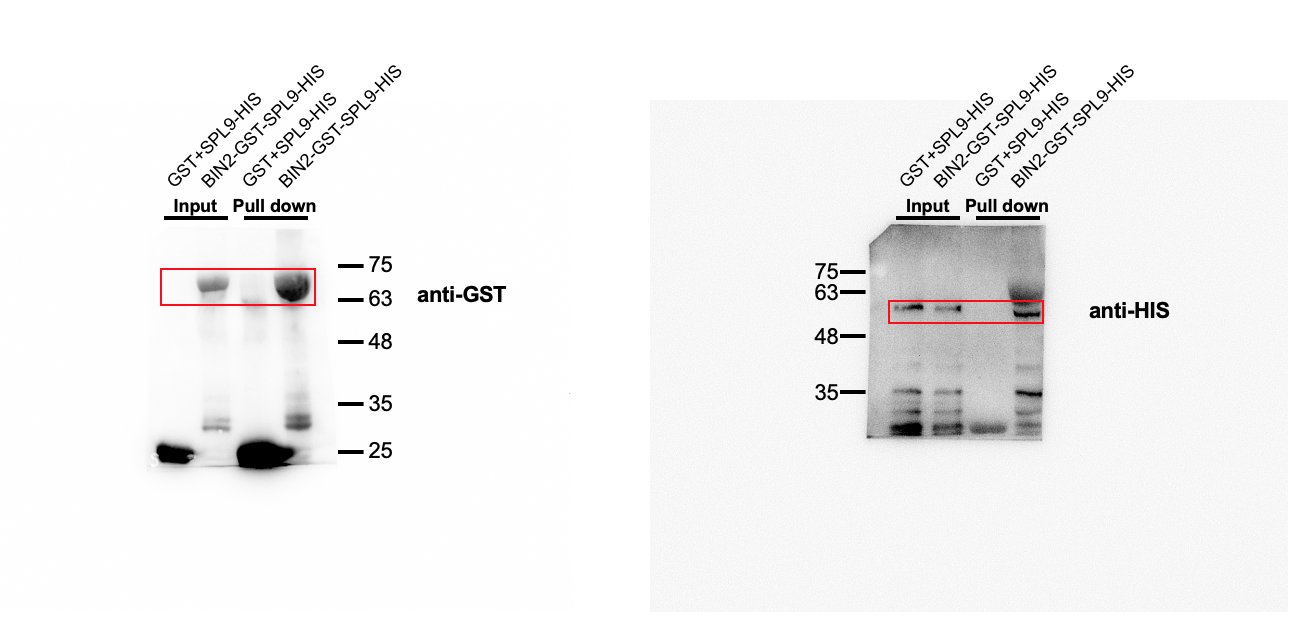


**Figure 4d**


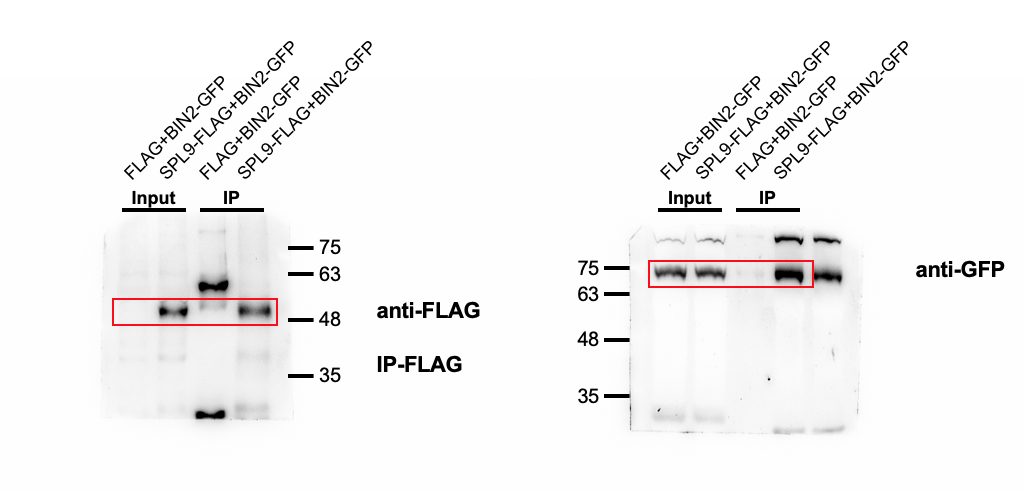


**Figure 5b**


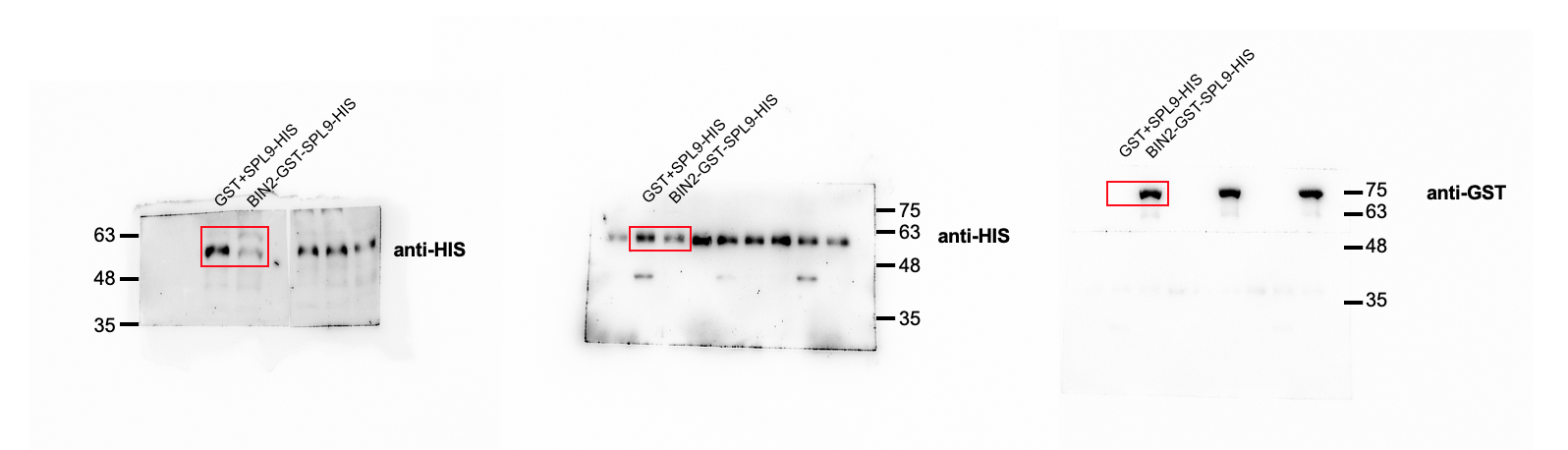


**Figure 5c**


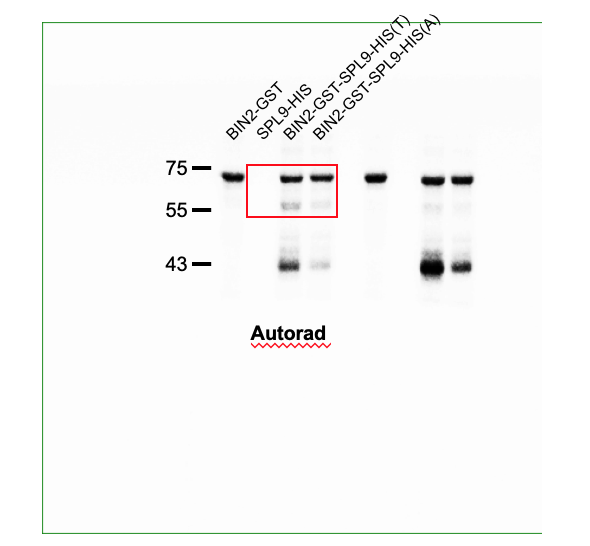


**Figure 5g**


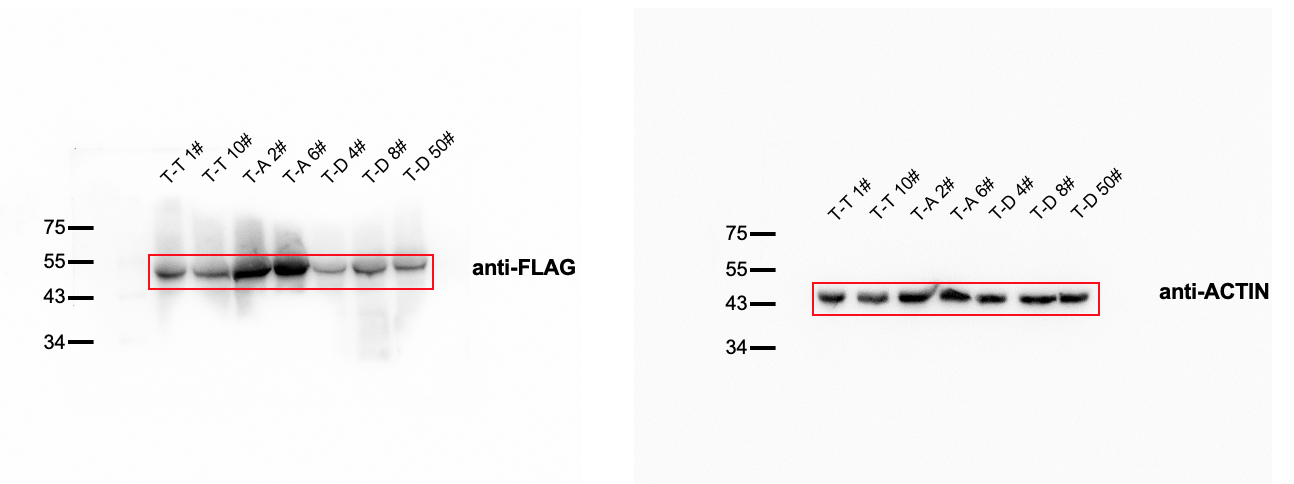


**Figure 6d**


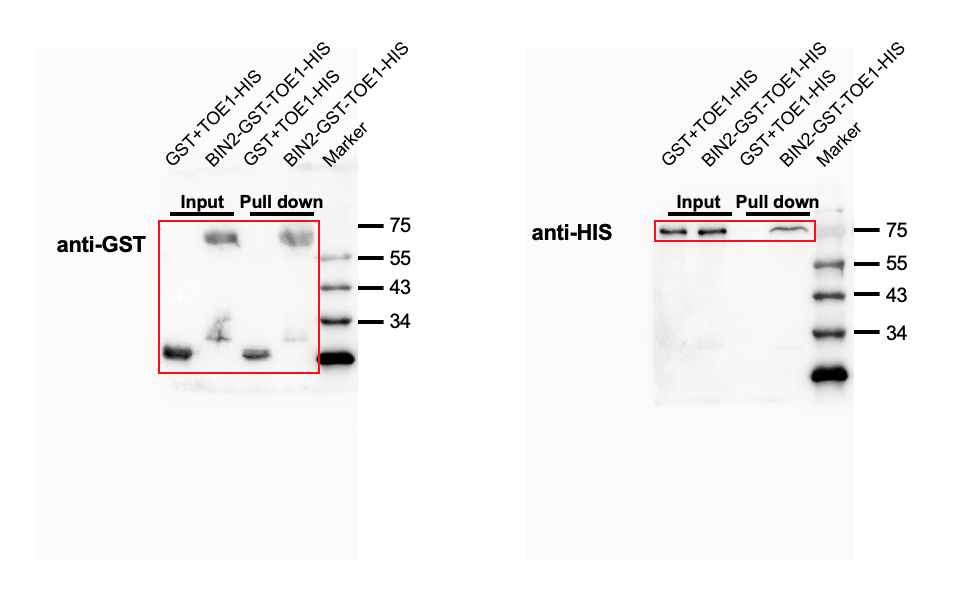


**Figure 6e**


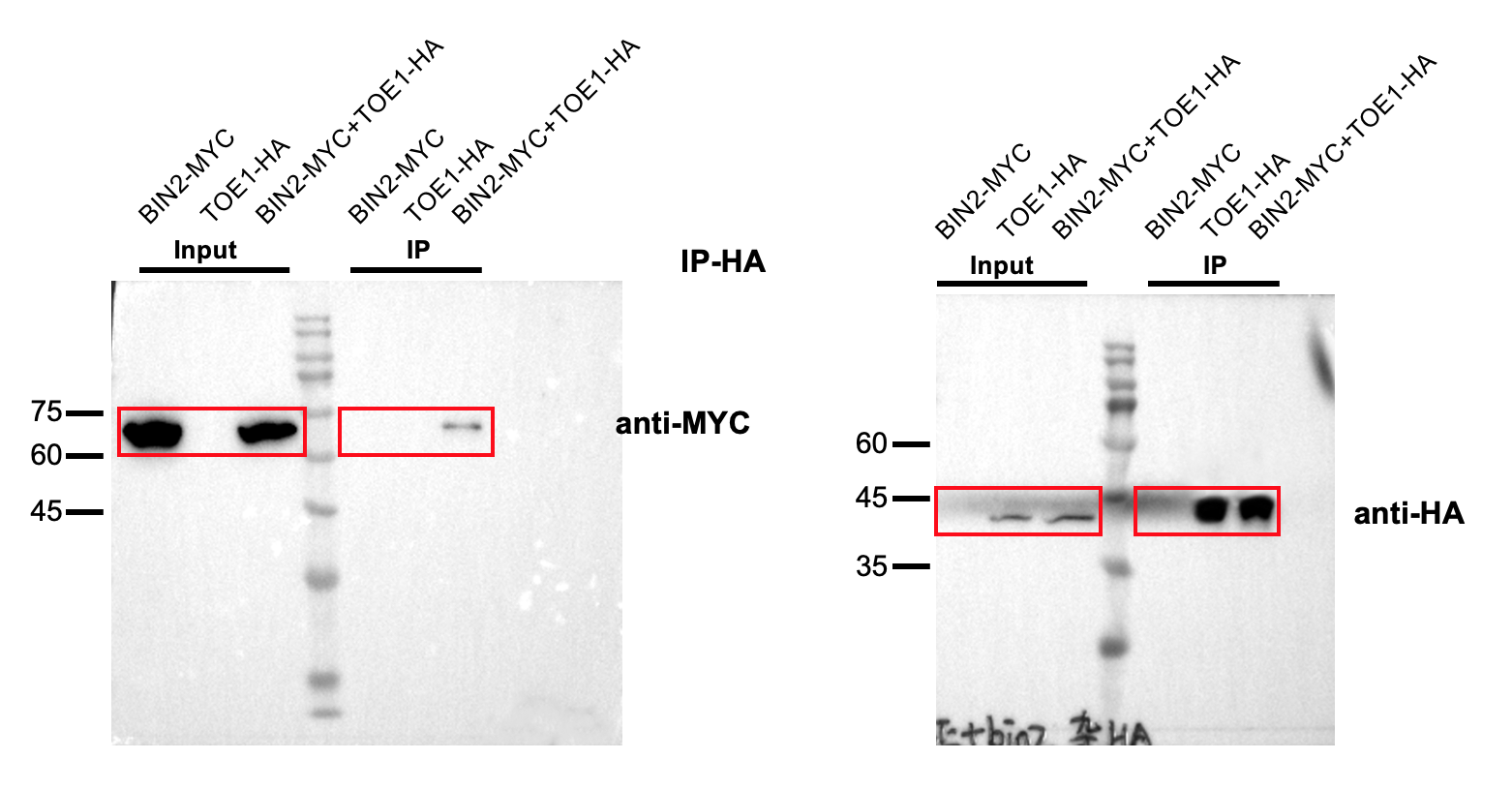


**Figure 7a**


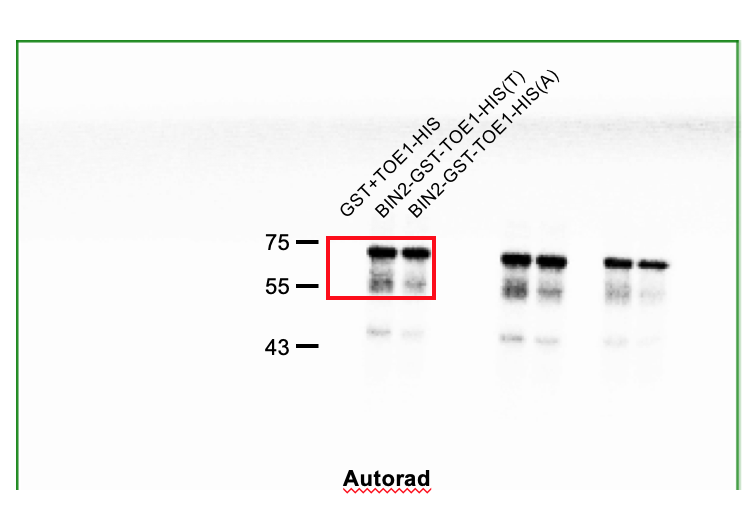


**Figure 7e**


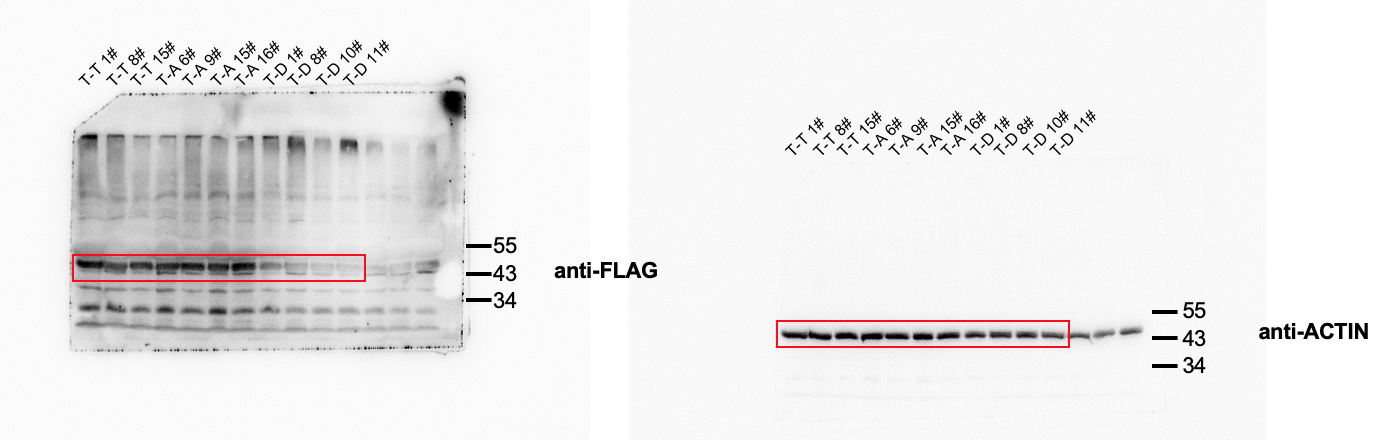


**Figure 8a**


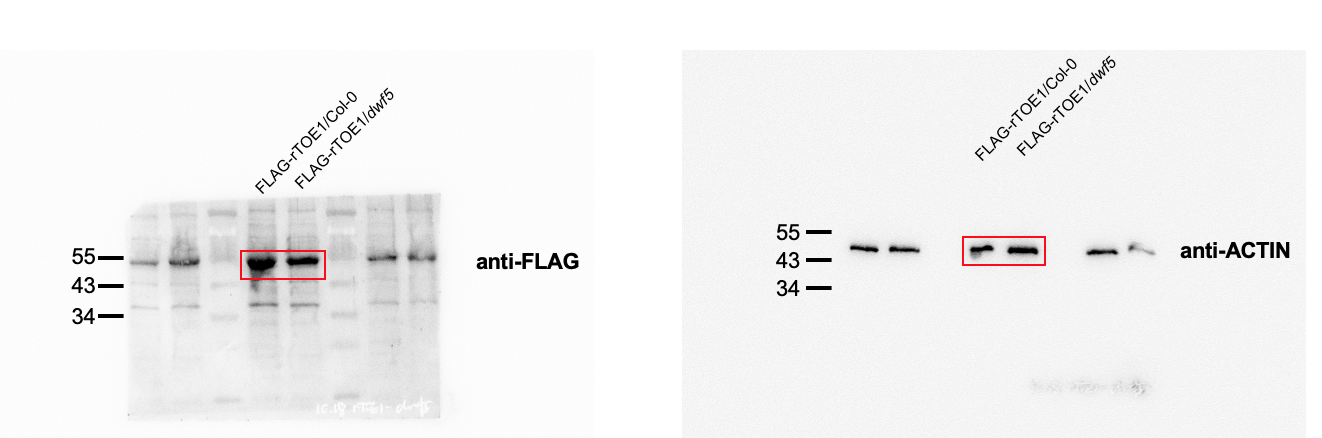


**Figure 8b**


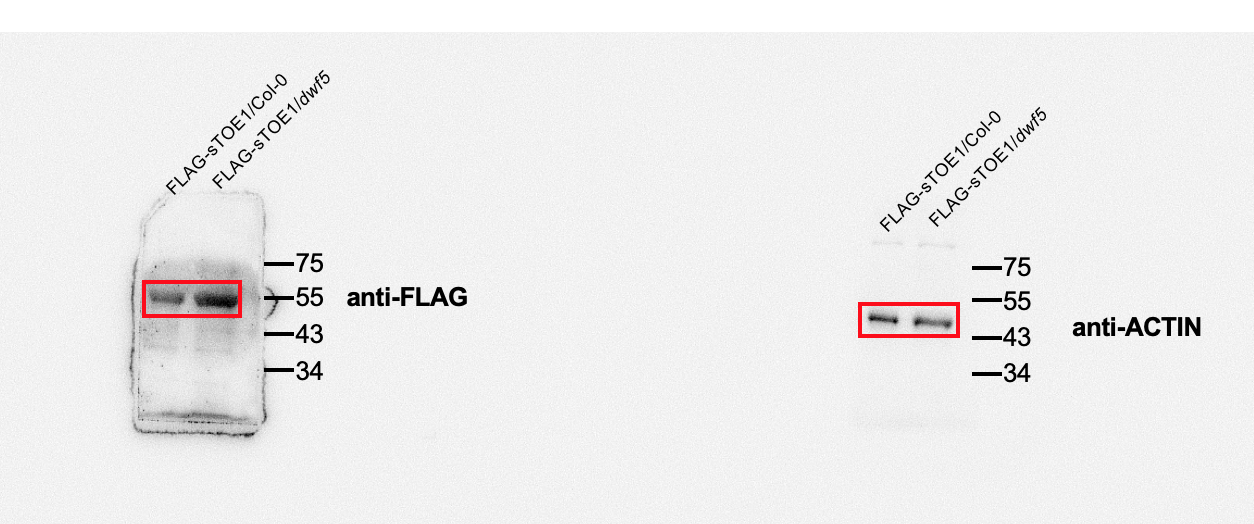


**Supplementary Figure 1a**


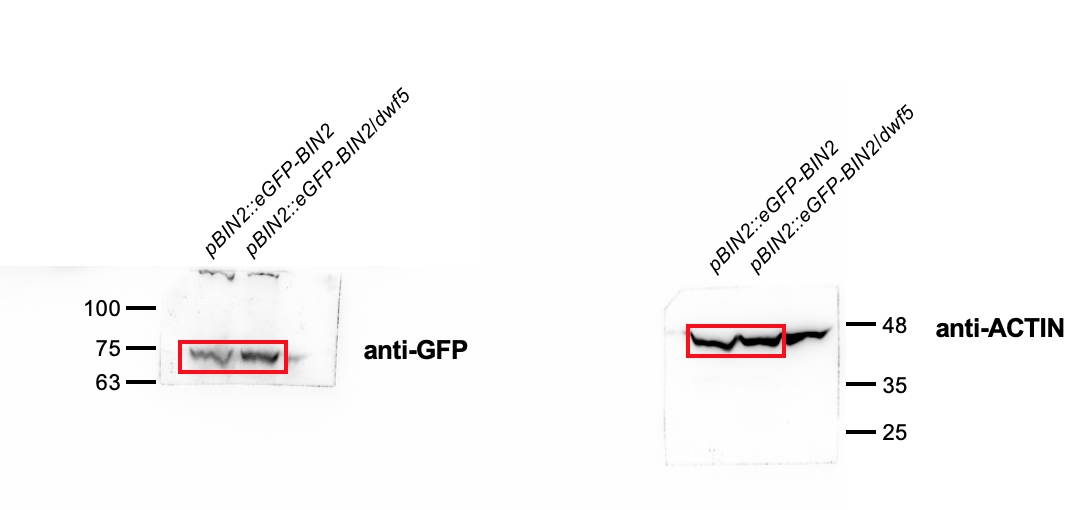


**Supplementary Figure 2b**


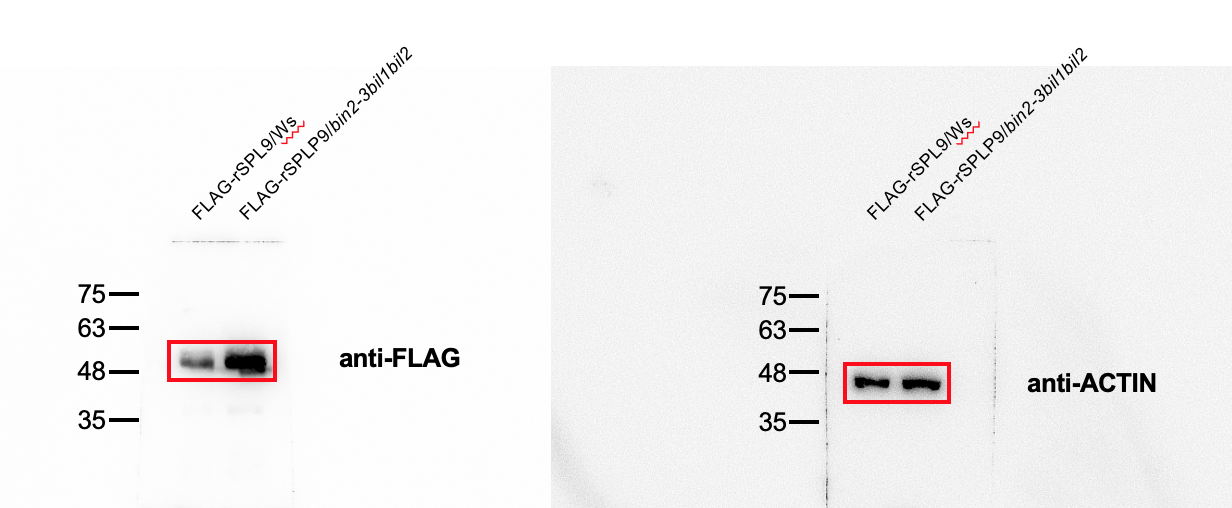


**Supplementary Figure 4b**


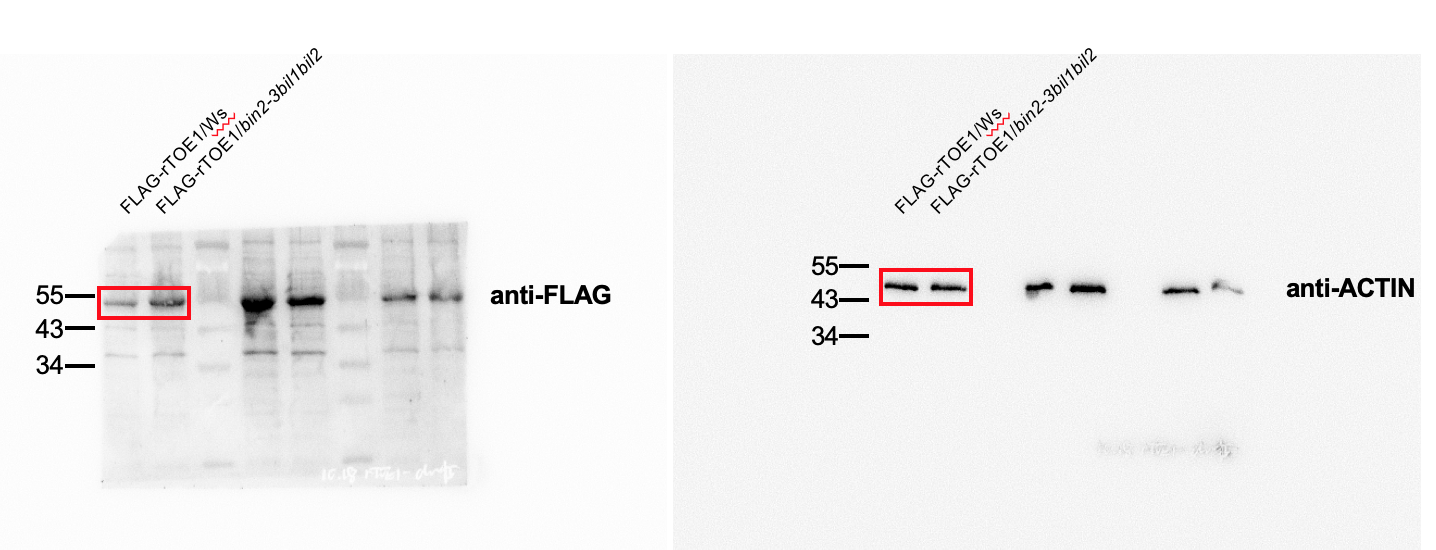


**Supplementary Figure 4c**


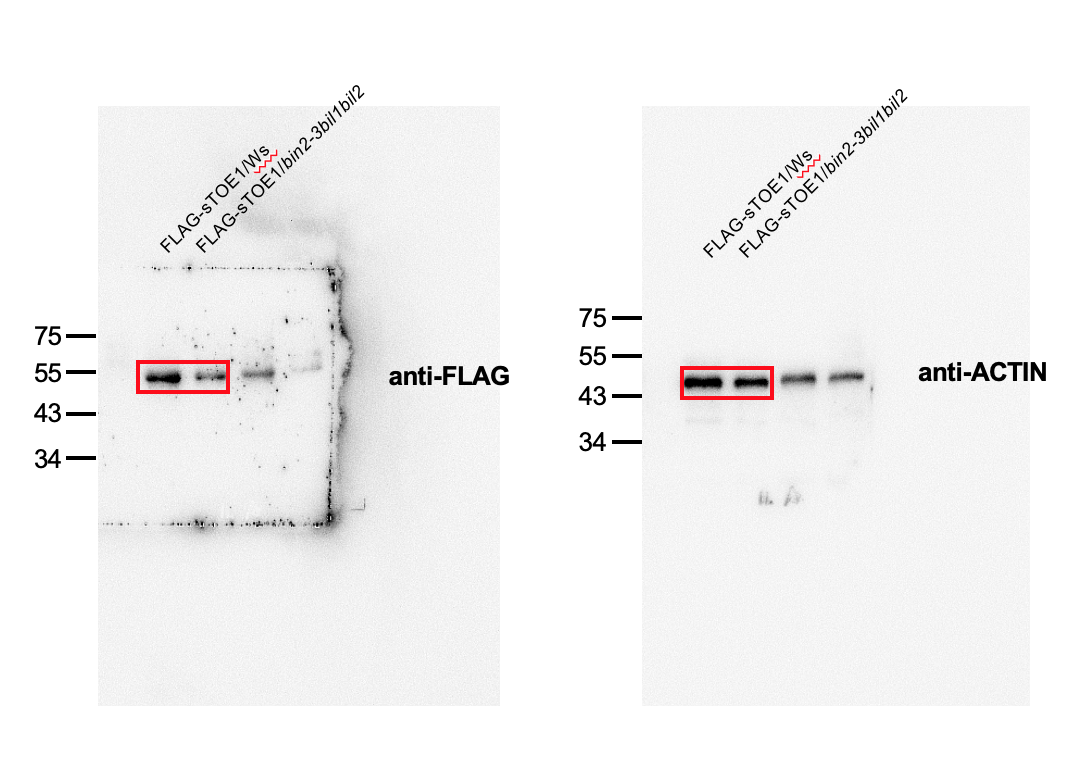

Supplement: Supplementary file 4 — Source data [file 41467_2023_38207_MOESM4_ESM.zip › Source Data/Gel.docx]
